# Investigation of the expression patterns and correlation of DNA methyltransferases and class I histone deacetylases in ovarian cancer tissues

**DOI:** 10.3892/ol.2012.1057

**Published:** 2012-12-03

**Authors:** YIFENG GU, PEIFANG YANG, QING SHAO, XIA LIU, SHENG XIA, MIAOMIAO ZHANG, HUAXI XU, QIXIANG SHAO

**Affiliations:** 1 Department of Immunology, School of Medical Science and Laboratory Medicine, Jiangsu University, Zhenjiang 212013;; 2 Department of Gynecology and Obstetrics, Affiliated Hospital of Jiangsu University, Zhenjiang 212001;; 3 Zhenjiang Key Laboratory of Medical Diagnosis, Zhenjiang 212001, Jiangsu, P.R. China

**Keywords:** ovarian cancer, DNA methyltransferases, histone deacetylases, expression pattern, correlation

## Abstract

Recent studies have reported that DNA methyltransferases (DNMTs) and histone deacetylases (HDACs) are involved in the epigenetic regulation of cancer, as well as promoting cell proliferation and tumorigenesis. These mechanisms also play important roles in ovarian cancer, but little is known concerning the correlation of DNMTs and HDACs in ovarian cancer. In the present study, we used quantitative real-time reverse transcription polymerase chain reaction (qRT-PCR) and immunohistochemical staining to examine the mRNA and protein expression of DNMTs and class I HDACs of tissues from 22 cases of ovarian cancer and 8 normal ovaries as a control. Furthermore, we assessed the correlation with clinicopathological stages and the mRNA expression of these genes. The results indicated that the mRNA expression of DNMT1, DNMT3b and class I HDACs was increased in ovarian cancers, while the expression of DNMT3a was not different between cancer tissues and normal ovaries. Additionally, the results of immunohistochemical staining demonstrated that DNMT1 and DNMT3b were significantly increased in ovarian cancer samples. Furthermore, the expression of DNMT1, DNMT3b, HDAC1 and HDAC2 was significantly higher in stage III/IV compared with stage I/II ovarian carcinomas. The expression of HDAC2 was positively correlated with HDCA1, HDAC3 and HDAC8, and DNMT1 was positively correlated with DNMT3b. Simultaneously, DNMT3b was correlated with HDAC1 and HDAC2. HDAC1 may upregulate the expression of DNMTs, but this requires confirmation by *in vitro* and *in vivo* experiments. The overall high rate of expression for class I HDACs, DNMT1 and DNMT3b suggested that these mRNAs should be explored as predictive factors in ovarian cancer. In addition, HDAC1, HDAC2 and DNMT3b cooperated in controlling ovarian cancer progression. Determining the correlations between HDACs and DNMTs in ovarian cancer will not only further clarify the mechanisms of genesis and development, but also guide clinical therapy using the inhibitors of HDACs and DNMTs.

## Introduction

Ovarian cancer is the most lethal gynecological malignancy and the fifth leading cause of cancer-related mortality among females, as well as the ninth most common cancer among females. Its incidence rate ranks third, following that of cervical and uterine cancer. Due to the lack of early symptoms, >70% of patients are diagnosed with advanced-stage disease, by which stage the cancer has spread into adjacent tissues and organs beyond the ovaries (1). Furthermore, 30–40% of patients will succumb to ovarian cancer even in the early stage. For these reasons, the mortality rate of ovarian cancer ranks first among the various tumors affecting females. The five-year survival rate of ovarian cancer is <20%, and has shown only modest improvement over the past 40 years (2). The most advanced stage ovarian cancer patients are sensitive to standard chemotherapies, but relapse occurs in over 70% of patients, resulting in chemoresistant, fatal disease (3). Therefore, a better understanding of the molecular pathogenesis of ovarian cancer will contribute to identifying novel biomarkers in the early stage, developing new therapeutic targets, and possibly increasing the five-year survival rate of ovarian cancer, thus saving the lives of many patients (4).

Epigenetic aberrations are common in the development and progression of cancer cells and are mediated by DNA methyltransferases (DNMTs), histone deacetylases (HDACs) and microRNA (miRNA). These modifications alter gene function and malignant cellular transformation. DNA methylation is a reversible reaction, catalyzed by three major DNMTs. One is DNMT1, which preserves the methylation patterns throughout each cell division (5,6). The others are DNMT3a and DNMT3b, which transfer a methyl group to previously unmethylated genomic regions (7). Methylation occurs in specific genomic areas called CpG islands (8,9), usually at the promoter region of a gene, and prevents gene expression.

Histone modifications, particularly acetylation and deacetylation, are the major driving force for epigenetic gene regulation. They may regulate gene transcription by regulating acetylation of DNA sequence-specific transcription factors. Examples include p53, E2F and Sp3, where deacetylation has been linked to reduced DNA binding or transcriptional activity (10–12). To date, 18 members of human HDACs have been identified and categorized into four classes, based on homology to yeast HDACs and phylogenetic analysis (13,14). In general, class I HDACs (HDACs 1–3 and 8) are primarily located in the nucleus and are associated with transcriptional repressors and cofactors.

Overexpression of DNMTs and HDACs has been reported in various types of cancers including ovarian cancer (15–21). Recently, a number of studies have demonstrated that DNMTs and HDACs contribute to the epigenetic regulation in cancer cells. The results of investigations by Zhou *et al*(22) and You *et al*(23) have suggested that HDACs may have the capacity to upregulate the expression of DNMTs. Fuks *et al* found that HDAC1 has the ability to bind DNMT1 and purify methyltransferase activity from nuclear extracts. Moreover, DNMT1 has a transcriptional repression domain, and directly recruits histone deacetylase activity (24). Clinical trials have demonstrated that DNMT and HDAC inhibitors may be effective reagents for cancer therapy (25,26). Therefore it is important to investigate the expression pattern and correlation of DNMTs and HDACs in cancer and to guide clinical anticancer therapy.

In our study, we investigated the expression levels of DNMTs and class I HDACs in ovarian cancer tissues with quantitative real-time reverse transcription polymerase chain reaction (qRT-PCR) and immunohistochemical staining, and analyzed the correlation of DNMTs and HDACs. The relevant mechanisms of DNMT and HDAC collaboration in ovarian cancer await further clarification.

## Materials and methods

### Antibodies and chemical reagents

Histostain™-Plus Kit (Cat. No. SP/9001) was a product of Zymed Laboratories Inc. (San Francisco, CA, USA) and purchased from Sizhengbai Biotech Company Ltd. (Beijing, China). Rabbit anti-human DNMT1 polyclonal antibody (Cat. No. ab19905) was purchased from Abcam plc. (Cambridge, UK). Rabbit anti-human DNMT3b polyclonal antibody (Q-25, Cat. No. sc-130740) was bought from Santa Cruz Biotechnology (Santa Cruz, CA, USA). TRIzol™ reagent was purchased from Life Technologies Co. (Shanghai, China). SuperScript™ one-step RT-PCR kit was bought from Toyobo (Shanghai) Bio Co., Ltd. (Shanghai, China). SYBR-Green mix reagent was a product of Takara Bio (Dalian) Co., Ltd. (Dalian, China).

### Patients and tissue samples

A total of 22 freshly resected ovarian cancer tissue samples and eight normal ovarian tissue samples (from ovariectomized patients suffering from other gynecological diseases) were collected at the Affiliated Hospital of Jiangsu University, Zhenjiang, China, in accordance with institutional guidelines and immediately frozen in liquid nitrogen for further analysis. The study was approved by Jiangsu University’s ethical review committee and informed consent for the use of tissues was obtained for all individuals. The histopathological diagnosis was based on WHO criteria; the samples were assigned a grade based on Gynecologic Oncology Group criteria (27) and staged according to the International Federation of Gynecology and Obstetrics system (FIGO) (28).

The mean ages of normal and cancer patients were 54 years (range, 36 to 70 years) and 59 years (range, 37 to 76 years), respectively. Stage breakdown was: 2 (9%) in stage I, 5 (22.7%) in stage II, 9 (40.9%) in stage III, and 6 (27.3%) in stage IV. The tumor histotype was serous carcinoma in 19 patients (86.4%) and mucinous carcinoma in 3 (13.6%).

### Total RNA extraction and qRT-PCR

RNA was extracted from frozen tissue samples using the TRIzol reagent (Life Technologies) according to the manufacturer’s instructions. The dissolved RNA was stored at −70°C before use. RNA quality was assessed with a NanoDrop1000 spectrophotometer (Eppendorf; AG, Hamburg, Germany). RT-PCR was carried out using a SuperScript™ one-step RT-PCR kit (Toyobo) according to the manufacturer’s instructions. cDNA was synthesized by utilizing an oligo (dT) primer from 1 *μ*g total RNA at 42°C for 20 min, followed by inactivation of the reverse transcriptase at 94°C for 5 min. The qRT-PCR was performed in a final volume of 20 *μ*l containing 10 *μ*l SYBR-Green mix reagent (Takara), sense and antisense primers (0.3 *μ*l, 10 mM), and cDNA (1 *μ*l) and DNA-free water (8.4 *μ*l). Primer sequences are listed in Table I. Each DNMT and HDAC value was normalized to the expression of β-actin. Values are presented as the mean ± standard deviation (SD) of triplicate measurements.

### Immunohistochemical staining

Formalin-fixed, serial paraffin-embedded ovarian cancer tissues were immunostained by the streptavidin-biotin-peroxidase complex (ABC) method, using a Histostain-Plus Kit (Zymed Laboratories Inc.). Briefly, 3-*μ*m-thick sections were placed on 3-aminopropyltriethoxysilane (APES)-coated slides, deparaffinized and rehydrated routinely. To unmask the antigen, sections were boiled in 0.01 M citrate buffer (pH 6.0) in a microwave oven for 20 min. For quenching of endogenous peroxidase activity, sections were treated with 0.3% hydrogen peroxide for 10 min at room temperature. After rinsing the sections in phosphate-buffered saline (PBS, pH 7.4), the nonspecific binding site was blocked with 10% normal goat serum for 20 min at room temperature. The blocking serum was discarded and then the primary antibodies were added directly. Rabbit anti-human DNMT1 polyclonal antibody (Abcam) was diluted to 1:100, rabbit anti-human DNMT3b polyclonal antibody (Santa Cruz Biotechnology) 1:50 in PBS, respectively. The sections were incubated at 4°C overnight. After rinsing in PBS, biotinylated goat anti-rabbit immunoglobulin G (IgG) (Zymed Laboratories Inc.) was added, and the sections were incubated for 30 min at room temperature. After washing in PBS, peroxidase conjugated streptavidin was added and incubated for 30 min at room temperature. While rinsing in PBS, the peroxidase reaction was performed using 0.02% 3, 3′-diaminobenzidine tetrahydrochloride (DAB) containing PBS and 0.15% hydrogen peroxidase for 3–15 min at room temperature. Finally, tissues were stained with Mayer’s hematoxylin, then dehydrated and mounted. Staining without primary antibody was used as a negative control.

### Statistical analysis

The data are expressed as the mean ± SD. Statistical analysis was performed with the Mann-Whitney U test between the normal group and tumor group, and we also used this statistical method in comparison of stage III/IV and stage I/II cases. Spearman’s rank correlation test was used to study correlations between different genes.

## Results

### mRNA expression of class I HDACs, DNMT1 and DNMT3b is upregulated in ovarian cancers

**Figure 1.**
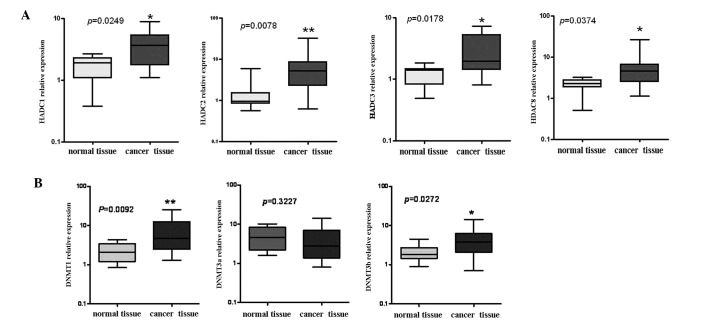
Relative levels of expression of (A) HDAC1, 2, 3 and 8, and (B) DNMT1, 3a and 3b mRNA by qRT-PCR in the ovarian cancer tissues (n=22), compared with the normal ovarian tissues (n=8). The level of expression for HDACs and DNMTs is normalized to β-actin. For comparison of expression data between different groups the Mann-Whitney U test was used. ^*^P<0.05, ^**^P<0.01. HDAC, histone deacetylase; DNMT, DNA methyltransferase; qRT-PCR, quantitative reverse transcription polymerase chain reaction.

The expression levels of the class I HDACs (HDAC 1, 2, 3 and 8), DNMT1, DNMT3a and DNMT3b transcripts were determined by qRT-PCR analysis and shown in Fig. 1. The relative expressions of HDAC 1, 2, 3 and 8 mRNA in ovarian cancers (n=22) were significantly higher than those in normal tissues (n=8), particularly for HDAC2 (P<0.01; Fig. 1A). Similarly, the relative expressions of DNMT1 and DNMT3b mRNA in ovarian cancers (n=22) were significantly higher than those in normal tissues (n=8), particularly for DNMT1 (P<0.01; Fig. 1B). The relative expression of DNMT3a was not different between the two groups (P=0.03227; Fig. 1B middle panel).

### Gene expression levels of DNMTs and HDACs were correlated with the FIGO stage

**Figure 2.**
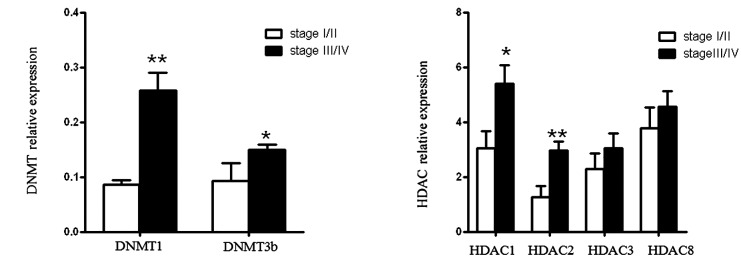
Positivity index for DNMT1 and 3b, and HDAC1, 3 and 8 in ovarian cancers according to the FIGO classification. Each value indicates the mean ± SD. ^*^P<0.05, ^**^P<0.01. DNMT, DNA methyltransferase; HDAC, histone deacetylase.

In accordance with FIGO stage, we divided the patients into two groups; stage I/II (n=7) and stage III/IV (n=15); and evaluated the mRNA levels at the different stages. Results showed that DNMT1 and DNMT3b expression was significantly increased in stage III/IV compared with stage I/II, particularly for DNMT1 (P<0.01; Fig. 2A). The relative expression of HDAC1 and HDAC2 demonstrated a similar result (Fig. 2B). In contrast, the levels of HDAC3 and HDAC8 were not different between the two groups (Fig. 2B).

### Protein expression levels of DNMT1 and DNMT3b were increased in ovarian cancers

**Figure 3.**
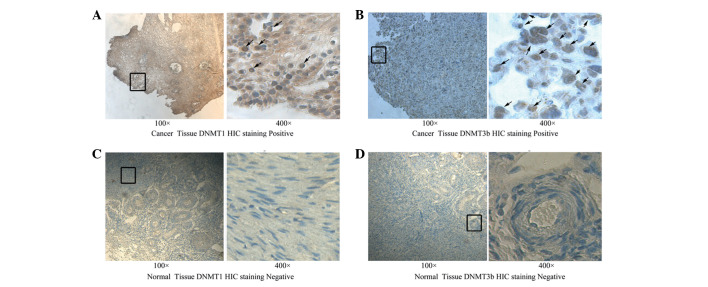
Immunohistochemical staining of DNMT1 and DNMT3b in ovarian tumor tissues and ovarian normal tissues. (A) Immunohistochemical staining for DNMT1 in ovarian tumor tissue, showing that some cancer cells expressed DNMT1 in nuclei (arrows indicate positive cells). (C) Immunohistochemical staining for DNMT1 in normal ovarian tissue showing no positive staining in cells. (B) Immunohistochemical staining for DNMT3b in ovarian tumor tissue showing that most of the cancer cells expressed DNMT3b in the nuclei (arrows indicate positive cells). (D) Immunohistochemical staining for DNMT3b in normal ovarian tissue showing no positive staining in cells. Magnification: Left images, ×100; Right images, ×400. DNMT, DNA methyltransferase.

The expression of DNMT1 and DNMT3b was assessed *in situ* on paraffin sections of normal ovarian tissues (n=8) and malignant ovarian tumors (n=22). Fig. 3 shows the representative immunohistochemistry results for DNMT1 and DNMT3b expression in tissues. The intensity of staining for DNMT1 and DNMT3b in malignant ovarian tumors was significantly greater than that in normal tissues (Fig. 3).

### Correlation among the mRNA expression of class I HDACs, DNMT1 and DNMT3b

We examined the correlation among the expression of class I HDACs, DNMT1 and DNMT3b. The expression of HDAC2 showed a significantly positive correlation with HDAC1 (ϱ= 0.4958, P=0.0309), HDAC3 (ϱ=0.4719, P=0.0413) and HDAC8 (ϱ=0.6123, P=0.0027). Similarly, there was a positive correlation between DNMT1 and DNMT3b (ϱ=0.4736, P=0.026). Unexpectedly, there was no correlation with HDAC1, HDAC3 and HDAC8. Notably, DNMT3b had a positive correlation with HDAC1 (ϱ=0.5158, P=0.0238) and HDAC2 (ϱ=0.4857, P=0.035; Table II). In addition, in most ovarian cancer patients the expression of the DNMTs were found to be upregulated, the expressions of HDACs are also upregulated; while the expressions of the HDACs are increased, but the expression of DNMTs are not as increased as expected (Table III).

## Discussion

Previous studies have confirmed the epigenetic regulation of the development and differentiation of the body. Epigenetic abnormalities often have a close correlation with the occurrence of a number of diseases, including cancer, alcoholic liver diseases, degenerative diseases of the nervous system, mental diseases, autoimmune diseases and cardiovascular diseases (29–31). It has been found that most cancers have abnormal DNA methylation, DNA deacetylation and miRNA expression, while the expression of DNMTs and HDACs is generally increased (32,33). In mammals, DNA methylation and deacetylation are mostly mediated by DNMT1, DNMT3a, DNMT3b and class I HDAC regulation. It has been indicated that HDACs and DNMTs have important regulatory roles in human breast and cervix cancer cells. Furthermore, the inhibition of HDACs downregulates the expression of DNMT1 (22,23). Ovarian cancer is one of the common malignant tumors in female reproductive organs and is associated with high expression of DNMTs and of HDACs (4,34,35), but there are no studies concerning the correlation between DNMTs and HDACs in ovarian cancer.

In this study, we describe a high-level expression of class I HDAC isoforms and two functional DNMTs in ovarian cancer which catalyze cytosine methylation, and may therefore be of importance in dysregulating gene expression, in particular that of tumor suppressor genes. We used qRT-PCR assays to study the mRNA expression of DNMTs and class I HDACs in a series of 22 ovarian cancers. Overexpression of HDACs results in repression of important growth suppressive genes in numerous cancer cells, and is an important mechanism to promote cancer cell proliferation (36). Weichert *et al* revealed that a high proportion of ovarian carcinomas demonstrated class I HDAC protein expression using a tissue microarray, and the expression of class I HDACs indicates poor prognosis (37). High expression levels for class I HDACs were assessed by immunohistochemistry (38,39), which is in line with our results. Compared with normal ovarian tissue, our results showed that HDAC2 was highly prominent (P<0.01). At the same time, the expression of HDAC2 was significantly higher at stage III/IV than at stage I/II (P<0.01). Overexpression of HDAC2 was reportedly correlated with a more advanced stage in gastric carcinoma and it was also a prognostic indicator for a poor outcome in cases of prostate carcinoma (16,21). Although these studies indicated that the increasing expression of HDAC2 was associated with tumor progression, our study is the first to demonstrate a clear correlation between HDAC2 expression and advanced stage ovarian cancer (Table II). On the other hand, DNA hypermethylation of tumor suppressor genes in the promoter region was frequently observed in hepatocellular carcinomas (40), DNMTs are possibly responsible for DNA hypermethylation of these genes since their expression levels are known to increase during early tumorigenesis (17). We found that the mRNA and protein expression levels of DNMT1 and 3b were high in ovarian cancers compared with normal tissues, while for DNMT3a there was no difference between them. The result was similar to those observed in breast cancer (41). DNMT1 was highly prominent (P<0.01) among these DNMTs. Furthermore, the expression of DNMT1 was significantly higher at stage III/IV than at stage I/II (P<0.01), supporting a role for DNMT1 in tumorigenesis (42). The mRNA expression of DNMT1, DNMT3b, HDAC1 and HDAC2 were particularly prominent in high-grade tumors in ovarian cancers, which indicated that they may be the biomarkers of tumor aggressiveness and proliferation, as their high expression is closely associated with ovarian carcinoma progression. In our results, the expression of HDAC2 was correlated with HDCA1, HDAC3 and HDAC8, and the expression of DNMT1 was correlated with DNMT3b. A notable aspect of our results is that the level of DNMT3b was correlated with HDAC1 and HDAC2 in ovarian cancer, which demonstrated that the two epigenetic events cooperated in controlling ovarian cancer progression (Table II). There have been reports that methylation of histone H3 lysine 9 may be triggered by DNA methylation (43), and DNMTs have also been shown to interact with HDACs, histone methyltransferases (HMTs) and methylcytosine-binding proteins in a complex network (22,23,44). Our results also imply that the expression of the DNMTs are regulated by HDACs (Table III); however, these findings need to be demonstrated by further research. This finding was in accordance with the research results of Zhou *et al*(22) and You *et al*(23).

In summary, our studies demonstrate that the mRNA expression of DNMT1, DNMT3b and class I HDACs was increased in ovarian cancers. HDAC1, HDAC2, DNMT1 and DNMT3b expression increased with stage. Furthermore, HDAC1, HDAC2 and DNMT3b cooperated in controlling ovarian cancer progression and the HDACs may upregulate the expression of DNMTs. Detecting the expressions of the DNMTs and HDACs may aid the diagnosis and guide the clinical use of the inhibitors of DNMTs and HDACs to treat ovarian cancer. Further exploration is warranted to gain more information.

## Figures and Tables

**Table I.** Characteristics of primers used for qRT-PCR.

Primer	Sequence (5′-3′)	Cycles	Annealing temp (°C)
β-actin (genebank no. NM-001101)	Sense: CACGAAACTACCTTCAACTCC		
	Antisense: CATACTCCTGCTTGCTGATC	25	57
HDAC1 (genebank no. NM-004964)	Sense: AACCTGCCTATGCTGATGCT		
	Antisense: CAGGCAATTCGTTTATCAGA	30	57
HDAC2 (genebank no. NM-001527)	Sense: GGGAATACTTTCCTGGCACA		
	Antisense: ACGGATTGTGTAGCCACCTC	35	60
HDAC3 (genebank no. NM-003883)	Sense: TGGCTTCTGCTATGTCAACG		
	Antisense: GCACGTGGGTTGGTAGAAGT	35	60
HDAC8 (genebank no. NM-001166418)	Sense: TTTTCCCAGGAACAGGTGA		
	Antisense: AGCTCCCAGCTGTAAGACC	35	57
DNMT1 (genebank no. NM-001130823)	Sense: CTACCAGGAGAAGGACAGG		
	Antisense: CTCACAGACGCCACATCG	30	62.5
DNMT3a (genebank no. NM-153759)	Sense: TATGAACAGGCCGTTGGCATC		
	Antisense: AAGAGGTGGCGGATGACTGG	35	63.5
DNMT3b (genebank no. NM-001207056)	Sense: TTGGGCATAAAGGTAGGAA		
	Antisense: CATACTCCTGCTTGCTGATC	35	57

β-actin gene was used as an internal standard to normalize the amount of total RNA present in each reaction. qRT-PCR, quantitative reverse transcription polymerase chain reaction; HDAC, histone deacetylase; DNMT, DNA methyltransferase.

**Table II.** Spearman’s correlations between the mRNA of class I HDACs, DNMT1 and DNMT3b.

Gene	Correlation analysis	HDAC1	HDAC2	HDAC3	HDAC8	DNMT1	DNMT3a	DNMT3b
HDAC1	Correlation coefficient	1.000	0.4958^a^	0.3361	−0.2027	0.2214	0.0542	0.5158^a^
	P-value		0.0309	0.0797	0.4052	0.3622	0.8105	0.0238
HDAC2	Correlation coefficient		1.000	0.4719^a^	0.6123^b^	−0.2784	0.4490	0.4587^a^
	P-value			0.0413	0.0027	0.2484	0.0361^a^	0.035
HDAC3	Correlation coefficient			1.000	−0.1659	−0.224	0.0616	−0.2767
	P-value				0.2487	0.3566	0.7854	0.2515
HDAC8	Correlation coefficient				1.000	0.2082	0.0311	−0.2047
	P-value					0.3924	0.8908	0.4007
DNMT1	Correlation coefficient					1.000	0.0734	0.4736^a^
	P-value						0.7454	0.026
DNMT3a	Correlation coefficient						1.000	0.0429
	P-value							0.8496
DNMT3b	Correlation coefficient							1.000
	P-value							

a P<0.05,

b P<0.01. HDAC, histone deacetylase; DNMT, DNA methyltransferase.

**Table III.** Relative expression of class I HDACs, DNMT1 and DNMT3b in 22 samples of ovarian cancer.

		The Ct values for ovarian cancer tissues/the Ct values for normal control tissues						
Sample	Stage	DNMT 1	DNMT 3a	DNMT 3b	HDAC1	HDAC2	HDAC3	HDAC8
1	I	0.75	0.69	1.59^a^	1.61^b^	0.37	0.77	1.42
2	I	1.27	1.18	1.60^a^	1.27	1.22	1.95^b^	2.09^b^
3	II	0.62	0.87	1.02	1.57^b^	0.55	2.82^b^	1.91^b^
4	II	0.89	0.73	0.89	0.64	1.80^b^	0.65	2.03^b^
5	II	1.72^a^	0.71	1.38	0.67	1.36	1.36	0.75
6	II	1.61^a^	1.13	1.87^a^	2.34^b^	2.04^b^	4.32^d^	8.02^f^
7	II	1.29	1.42	1.42	0.89	0.90	1.58^b^	1.12
8	III	1.43	0.98	0.93	1.80	1.60	0.77	1.36
9	III	1.63^a^	0.63	4.64^c^	4.99^d^	0.82	0.82	3.12^d^
10	III	1.81^a^	0.70	1.34	0.97	1.02	1.59^b^	2.78^b^
11	III	3.37^c^	0.70	3.58^c^	5.33^d^	2.05^b^	1.14	2.41^b^
12	III	14.6^e^	1.22	8.62^e^	5.05^d^	8.57^f^	2.28^b^	1.74^b^
13	III	3.10^c^	0.68	1.72^a^	2.78^b^	2.55^b^	4.76^d^	3.08^d^
14	III	1.01	0.81	5.14^c^	0.94	1.69^b^	3.26^d^	0.73
15	III	1.68^a^	1.27	18.12^e^	3.65^d^	13.21^f^	5.60^d^	3.11^d^
16	III	3.58^c^	1.34	1.32	2.50^b^	2.10^b^	1.46	1.81^b^
17	III	2.24^a^	1.14	3.68^c^	1.40	3.70^d^	2.03^b^	2.17^b^
18	IV	3.11^c^	2.16^a^	3.64^c^	3.05^d^	5.85^d^	1.72^b^	2.49^b^
19	IV	1.70^a^	2.18^a^	1.68^a^	1.84^b^	5.48^d^	0.65	10.8^f^
20	IV	1.55^a^	0.76	0.95	0.64	1.55^b^	1.06	2.45^b^
21	IV	4.61^c^	0.57	4.40^c^	2.06^b^	2.60^b^	4.26^d^	2.68^b^
22	IV	2.50^a^	1.32	3.12^c^	1.94^b^	3.40^d^	0.55	4.60^d^

a Increase in DNMT expression >1.5- but ≤3-fold;

b Increase in HDAC expression >1.5- but ≤3-fold;

c Increase in DNMT expression >3- but ≤6-fold;

d Increase in HDAC expression >3- but ≤6-fold;

e Increase in DNMTs expression >6-fold;

f Increase in HDACs expression >6-fold. HDAC, histone deacetylase; DNMT, DNA methyltransferase.
